# Hereditary Hemorrhagic Telangiectasia and Arterio-Venous Malformations—From Diagnosis to Therapeutic Challenges

**DOI:** 10.3390/jcm11092634

**Published:** 2022-05-07

**Authors:** Mariana Floria, Elena Diana Năfureanu, Diana-Elena Iov, Oana Sîrbu, Mihaela Dranga, Anca Ouatu, Daniela Maria Tănase, Oana Bogdana Bărboi, Vasile Liviu Drug, Mihail Dan Cobzeanu

**Affiliations:** 1 Internal Medicine Department, “Grigore T. Popa” University of Medicine and Pharmacy, 700115 Iași, Romania; floria.mariana@umfiasi.ro (M.F.); diananafureanu@outlook.com (E.D.N.); dr.oana.sirbu@gmail.com (O.S.); mihaela_dra@yahoo.com (M.D.); anca.ouatu@gmail.com (A.O.); oany_leo@yahoo.com (O.B.B.); vasidrug@email.com (V.L.D.); 2 “Dr. Iacob Czihac” Military Emergency Hospital, 700483 Iași, Romania; 3 Sf. Spiridon Emergency Hospital, 700111 Iași, Romania; cobzeanu_dan@yahoo.com; 4 Surgical Department, “Grigore T. Popa” University of Medicine and Pharmacy, 700115 Iași, Romania

**Keywords:** hereditary hemorrhagic telangiectasia, Osler-Weber-Rendu disease, arterio-venous malformations, pulmonary artery, embolization therapy, interventional

## Abstract

Hereditary hemorrhagic telangiectasia is a rare autosomal dominant vascular disease defined by the presence of mucosal and cutaneous telangiectasia and visceral arterio-venous malformations. The latter are abnormal capillary-free direct communications between the pulmonary and systemic circulations with the following consequences: arterial hypoxemia caused by right-to-left shunts; paradoxical embolism with transient ischemic attack or stroke and brain abscess caused by the absence of the normally filtering capillary bed; and hemoptysis or hemothorax due to the rupture of the thin-walled arterio-venous malformations (particularly during pregnancy). It is frequently underdiagnosed, commonly presenting as complications from shunting through arterio-venous malformations: dyspnea, chronic bleeding, or embolism. Arterio-venous malformations are present not only in the lungs, but can also be found in the liver, central nervous system (mainly in the brain), nasal mucosa, or the gastrointestinal tract. The first choice of therapy is embolization of the afferent arteries of the arterio-venous malformations, a minimally invasive procedure with a high efficacy, a low morbidity, and low mortality. Other therapeutic modalities are surgery (resection) or stereotactic radiosurgery (using radiation). Routine screening for arterio-venous malformations is indicated in patients diagnosed with this condition and can prevent severe complications such as acute hemorrhages, brain abscesses, or strokes. Clinicians should provide a long-term follow-up for patients with arterio-venous malformations, in an effort to detect their growth or reperfusion in case of previously treated malformations. In spite of two experts’ consensuses, it still possesses multiple therapeutic challenges for physicians, as several aspects regarding the screening and management of arterio-venous malformations still remain controversial. Multidisciplinary teams are especially useful in complex cases.

## 1. Introduction

Osler–Weber–Rendu disease, known as hereditary hemorrhagic telangiectasia (HHT), is a rare autosomal dominant vascular disease defined by the presence of cutaneous and mucosal telangiectasia and visceral arterio-venous malformations (AVMs). The prevalence of HHT according to studies conducted in Europe and Asia is estimated at approximately 1:5000 persons worldwide, although an analysis using US health insurance databases detected a much lower incidence of 0.3 per 10.000 persons [1]. A potential explanation for these varying results could reside in the insufficient knowledge regarding this disease and the lack of diagnosis. It was estimated that circa 500,000 to 1,000,000 people worldwide present with clinical manifestations of HHT [2].

## 2. Hereditary Hemorrhagic Telangiectasia—Diagnosis

The main underlying pathology in patients with HHT is a primary defect of the vascular network, characterized by a lack of intervening capillaries between arteries and veins. At a genetic level, mutations in multiple genes of the transforming growth factor-beta (TGF-β) signaling pathway are involved, normally responsible for regulating cell proliferation, migration, differentiation, and apoptosis [3]. Some studies have demonstrated that certain subtypes of HHT might be caused either by haploinsufficiency, involving different mutations that facilitate endoglin loss [4], or by gene alterations that could result in changes in endoglin protein structure [5].

**Figure 1:**
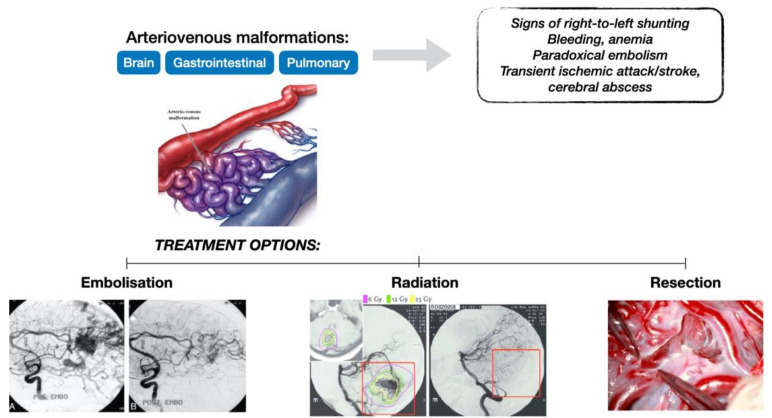
Arterio-venous malformations: from clinical consequences to treatment modalities.

Visceral AVMs are abnormal capillary-free direct communications between the pulmonary and systemic circulations with the following consequences (Figure 1):

- Arterial hypoxemia caused by right-to-left shunts;
- A paradoxical embolism with transient ischemic attack or stroke, and brain abscess caused by the absence of normally filtering capillary bed;
- Chronic bleeding like hemoptysis or hemothorax due to the rupture of the thin wall of the AVMs.

### 2.1. Classification

There are three subtypes of HHT described [2]:

- Type 1 is associated with an *ENG* gene mutation, encoding endoglin, and involves a higher number of pulmonary and central nervous system vascular malformations.
- Type 2 is associated with activin A receptor type II—like 1 (*ACVRL1*) gene mutations and with a higher incidence of primary pulmonary hypertension and hepatic manifestations of the disease. These two types are found in approximately 85% of cases referred to genetic testing for clinical suspicion of HHT [2].
- Type 3 is a subset linked to chromosome 5q31, but the gene has not been identified [6].

Additionally, other gene mutations that can cause HHT have been studied: protein tyrosine phosphatase, non-receptor type 14 (*PTPN14*), and *ADAM 17* are associated with pulmonary AVMs. Mutations in the *SMAD4* gene, which is normally an essential effector of the aforementioned TGF-β signaling pathway, are detected in less than 2% of cases with clinical suspicion of HHT and determine an increased risk of juvenile polyposis syndrome (juvenile polyposis/HHT overlap syndrome) [6]. Another possible genetic alteration is represented by *GDF2* mutations, encoding the activin receptor-like kinase-1-ligand BMP9, which have only been reported in a few cases so far [7]. These mutations are linked to the TGFβ signaling pathways, leading to AVMs mostly presenting in the lungs, liver, and brain. In addition to these, recent studies have reported a potential pathogenic role of mutations in the *RASA1* gene and suggest that their inclusion in molecular screening for HHT patients without *ENG*, *ACVRL1*, or *SMAD4* mutations could aid in a correct diagnosis [8].

A distinct genetic disorder associated with AVMs formation is the capillary malformation-arteriovenous malformation syndrome, which is defined by multiple oval capillary malformations with a surrounding white halo and synchronous AVMs.

According to the anatomic structure, pulmonary AVMs are classified in:

- simple (one artery supplying an aneurismal communication with a single draining vein) and
- complex (two or more arterial branches with two or more draining veins).

### 2.2. Clinical Diagnosis

Even if it is an autosomal dominant inherited disease, its manifestations are not typically present from birth, but more commonly develop with increasing age. The most frequent manifestation, epistaxis, is present in approximately 50% of affected individuals from the age of 10 and in 80–90% by the age of 21 [3]. After that, the AVMs become apparent from puberty and mucosal, cutaneous, and gastrointestinal telangiectasia appear progressively with age [9]. Although very rare, there are a few reports of infants and young children affected by HHT who exhibited characteristic clinical symptoms, such as nosebleeds, intracranial hemorrhage or even brain AVMs [10].

A clinical diagnosis of hereditary hemorrhagic telangiectasia is established based on the Curaçao criteria, family history (first degree relatives already diagnosed with this disease) [11,12] or by identification of a causative mutation. These criteria have good diagnostic performance and genetic testing is particularly helpful in patients with a suspected diagnosis of HHT. The interpretation of these criteria for HHT diagnosis is as follows: the presence of ≥three criteria results in a definite diagnosis, two criteria suggests that HHT is possible or suspected, and for less than two criteria diagnosis is unlikely.

The **Curaçao criteria** are represented by:

- **Telangiectasia**: these are pink to red lesions, usually 0.5–1.0 mm in diameter, which may appear at any site, especially on the face, nose, fingertips, lips, tongue, oral, and gastrointestinal mucosa. These lesions blanch when pressure is applied and refill immediately after release. Given their thin walls and close situation to the surface of the skin and mucosa, they are predisposed to rupture and bleeding [13].
- **Epistaxis:** the most prevalent symptom in HHT is epistaxis related to nasal telangiectasia, which can be spontaneous or recurrent and in severe cases can lead to anemia.
- **Visceral lesions:** represented by AMVs that may arise in various organs, with characteristic clinical presentations. Gastrointestinal lesions are usually evaluated by upper or lower digestive endoscopies and may become symptomatic through massive hemorrhages or anemia [7]. Pulmonary AVMs can be asymptomatic or may present with bleeding (hemoptysis, hemothorax) or signs of right-to-left shunting such as hypoxemia, cyanosis, poor exercise tolerance, dyspnea, orthodeoxia (decrease in oxygen saturation in an upright posture), nail clubbing, polycythemia, pulmonary embolism, heart failure, and pulmonary hypertension [14]. Neurological symptoms resulting from right-to-left shunting with embolic or infected material through pulmonary AVMs are migraines, transient ischemic attacks, strokes, seizures, and cerebral abscesses. The annual rate for stroke calculated by a meta-analysis is 0.92% and 0.32% for brain abscess [2]. Lesions in the liver may lead to congestive heart failure, portal hypertension, liver failure, and subsequent encephalopathy, and can be evaluated either by Doppler US or CT scan [7]. In the central nervous system, AVMs may be present in the brain or spinal cord and may determine cerebral abscesses, transient ischemic attacks, or even ischemic strokes [7]. These lesions can be examined by angiography or MRI scan.
- **Family history:** first degree relatives affected by the disease.

Pulmonary AVMs are thin-walled abnormal vessels, usually resulting in sac-like structures that replace normal capillaries between the pulmonary arterial and venous circulations. These are frequently multifocal, bilateral, and located predominantly at the bases of the pulmonary lobes, providing direct communication between pulmonary and systemic circulations. They tend to gradually increase in size over time; puberty, pregnancy, and pulmonary arterial hypertension may be contributing factors to this phenomenon [15]. A recent questionnaire-based study evaluating the level of awareness on the disease and its potential complications during pregnancy in women with a known HHT diagnosis suggests that more effort should be directed towards appropriate counselling, as more than 90% of the subjects had not been properly informed about the possible pregnancy-related risks [16]. In patients with severe disease, in which the majority of the lower lobe segmental arteries contain pulmonary AVMs, the incidence of neurological complications is greater compared to those with discrete pulmonary AVMs [14].

A recent multi-center study has found that the presence of symptomatic liver AVMs and gastrointestinal bleeding are predictors of mortality in HHT patients, irrespective of age. Intriguingly, brain and pulmonary AVMs were not significantly associated with mortality rates in the study population [17].

The risk and severity of AVMs might be assessed by Schobinger’s clinical staging [18]:

- Stage I (latency or inactivity): localized hyperemia. Pediatric patients can remain stable for long time in this stage if AVMs are small.
- Stage II (expansion): increasing of arterio-venous shunting.
- Stage III (progression): onset of symptoms and signs, such as pain, bleeding, or ulceration.
- Stage IV: large AVMs with a risk of cardiac involvement and decompensation.

Current recommendations state that in asymptomatic children of a parent affected by the disease, a HHT diagnosis should be suspected, unless clearly excluded by genetic testing [19].

### 2.3. Paraclinical Diagnosis

**Figure 2:**
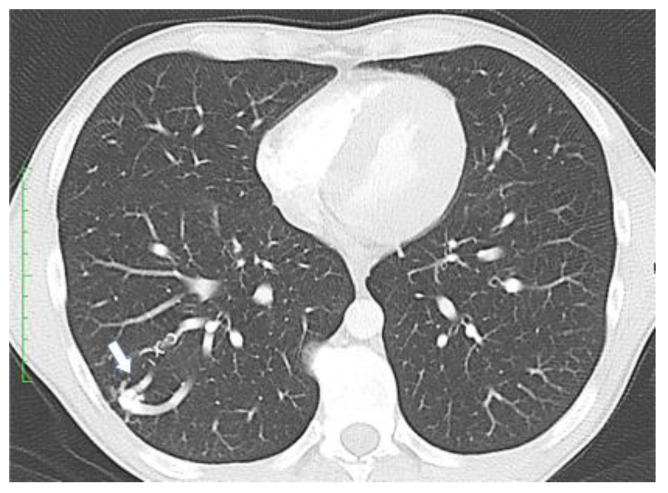
Chest computer tomography showing a pulmonary arterio-venous malformation (arrow).

**Figure 3:**
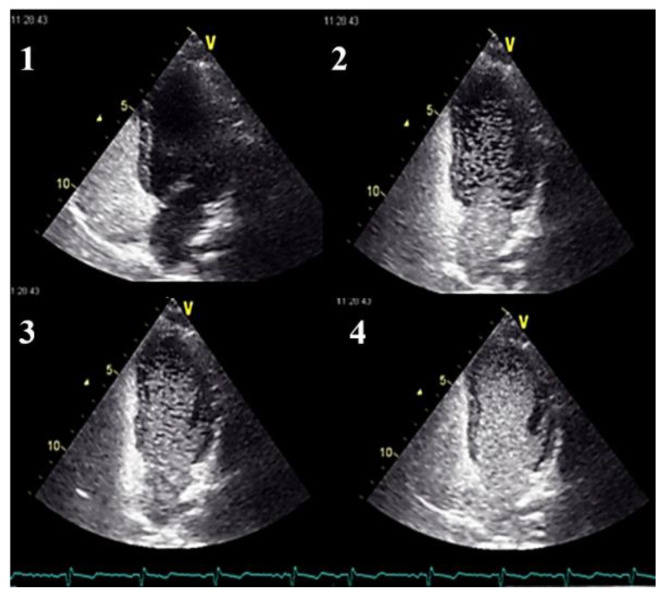
Contrast echocardiography showing progressive opacification with micro-bubbles of the left cavities in images 1 through 4, due to right-to-left intrapulmonary shunting. See also Supplementary Material—Video S1.

At the time of stroke and brain abscess, pulmonary AVMs can be clinically silent, so it is very important for these to be detected before becoming symptomatic. As chest X-ray and blood oxygen levels are not sensitive enough to detect the pulmonary AVMs, in this this regard, one must use a chest CT scan (Figure 2), magnetic resonance imaging, a pulmonary angiography, or contrast echocardiography [20]. Contrast echocardiography uses intravenous injections of micro-bubbles (Figure 3; Supplementary Materials: Video S1). Normal pulmonary capillary beds remove these bubbles, but in the presence of right-to-left shunting, these can be detected in the left side of the circulation.

Transthoracic contrast echocardiography should be used as the initial screening test for pulmonary AVMs [21]. In intracardiac shunts, bubbles appear right away, but there is a delay of 5–10 cardiac cycles in the presence of AVMs. The severity of the shunting can be evaluated by the number of micro-bubbles that appear on a single frame. Even though this method has the advantage of being widely available, easily repeatable, and without side effects, a CT scan should be performed in order to establish the possibility of embolization.

In adult patients with a definite or suspected HHT diagnosis, screening for liver AVMs should be performed [2]. In the presence of signs or symptoms suggestive of complicated liver AVMs, such as abnormal liver function tests, abdominal pain, portal hypertension, encephalopathy, pulmonary hypertension, heart failure, or abnormal levels of cardiac biomarkers, diagnostic testing should be performed using a Doppler ultrasound, a multiphase contrast CT scan, or contrast abdominal magnetic resonance imaging [19].

In pregnant women, the management of pulmonary AVMs is recommended as follows [19]:

- in the absence of symptoms, pulmonary AVMs screening should be performed initially, either by an agitated saline transthoracic contrast echocardiography or by a low-dose chest CT without contrast, according to local availability and expertise. When opting for a chest CT examination, it should ideally be performed early in the second trimester.
- in cases presenting with symptoms indicative of pulmonary AVMs, diagnostic testing by a low-dose non-contrast chest CT scan should be performed. This examination can be carried out at any gestational age, as clinically indicated.
- treatment of pulmonary AVMs is recommended starting with the second trimester, in the absence of other clinical contraindications.

## 3. Hereditary Hemorrhagic Telangiectasia—Therapeutic Challenges

Novel evidence regarding issues previously addressed in the first International HHT Guidelines for Diagnosis and Management [21] have been highlighted in the updated Second International HHT Guidelines [19]. In addition to that, anemia and anticoagulation, pediatric care, pregnancy, and delivery are newly addressed topics in this pathology. The summary of current recommendations is provided below:

**Management of liver AVMs:**

- Screening for liver AVMs should be performed in adults with definite or suspected HHT.
- In HHT patients with signs or symptoms suggestive of complicated liver AVMs, such as abnormal liver function tests, abdominal pain, portal hypertension, encephalopathy, pulmonary hypertension, heart failure, or abnormal levels of cardiac biomarkers, diagnostic testing should be performed using a Doppler ultrasound, a multiphase contrast CT scan, or contrast abdominal magnetic resonance imaging.
- An intensive first-line approach should only be instated in cases presenting with complicated or symptomatic liver AVMs, adapted to the specific type of complication.
- HHT cases that exhibit high-output cardiac failure and pulmonary hypertension should be managed multidisciplinarily by the Center of Excellence for HHT and a HHT cardiologist or a pulmonary hypertension specialty clinic.
- Clinicians should evaluate liver AVMs prognosis using available predictors in an effort to select patients in which closer monitoring may be advised.
- Intravenous administration of bevacizumab should be taken into consideration in cases with symptomatic high-output cardiac failure caused by liver AVMs with an insufficient response to first-line treatment.
- A referral to evaluation for liver transplantation is recommended in cases of refractory high-output cardiac failure, biliary ischemia, or complicated portal hypertension that arise as complications of liver AVMs.
- In patients with a proven or suspected HHT diagnosis, liver biopsy should be avoided.
- Given the fact that hepatic artery embolization is a temporising intervention with significant morbidity and mortality, it should not be performed in cases with liver AVMs.

**Management of pulmonary AVMs:**

- Clinicians should screen all patients with a suspected or confirmed HHT diagnosis for pulmonary AVMs.
- Clinicians should use transthoracic contrast echocardiography as the initial screening test for pulmonary AVMs.
- Clinicians should treat pulmonary AVMs with transcatheter embolotherapy.
- Clinicians should provide the following long-term recommendations to patients with diagnosed pulmonary AVMs (irrespective of previous treatment):
  1. In the event of procedures with risk of bacteremia, antibiotic prophylaxis should be done;
  2. When intravenous access is in place, special care should be taken in order to avoid intravenous air;
  3. SCUBA diving should be avoided.
- Long-term follow-up should be provided in cases with pulmonary AVMs, in an effort to diagnose the possible growth of untreated AVMs or the reperfusion of AVMs that have undegone treatment.

**Management of brain AVMs:**

- Magnetic resonance imaging as a screening method for brain AVMs in patients with suspected or definite HHT should be performed using a protocol both with and without contrast administration and implementing sequences that detect blood products, for sensitivity optimization.
- In cases of acute hemorrhage arrising as a complication of brain AVMs, definitive treatment should be recommended in a specialized center with neurovascular expertise.
- In all other patients diagnosed with brain AVMs, invasive testing and individualized management should be recommended in a center with neurovascular expertise.
- In pregnant women with possible or confirmed HHT with concurrent asymptomatic brain AVMs, definitive treatment of the brain AVMs should ideally be postponed until after delivery (which should follow obstetrical indications).

**Management of epistaxis:**

- In HHT-related epistaxis, moisturizing topical therapies that humidify the nasal mucosa should be used.
- Oral administration of tranexamic acid should be considered in cases of epistaxis that do not resolve with the use of moisturizing topical therapies.
- Ablative therapies such as laser treatment, radiofrequency ablation, electrosurgery, and sclerotherapy should be considered for the treatment for nasal telangiectasias, in cases that are non-responsive to moisturizing topical therapies.
- Systemic antiangiogenic agents should be considered for the management of epistaxis that has failed to resolve with any of the aforementioned therapies.
- Septodermoplasty or nasal closure should be taken into consideration in cases of epistaxis with insufficient improvement after topical application of moisturising therapies, ablative procedures and/or administration of tranexamic acid.
- Physicians should recommend the topical use of nasal mucosa humidifying agents for HHT-related epistaxis prophylaxis.
- Patients with HHT-related epistaxis who are willing to undergo treatment should be referred to otorhinolaryngologists with HHT expertise.
- If nasal surgery is considered for reasons other than epistaxis, an evaluation should be performed by an otorhinolaryngologist with expertise in HHT-related epistaxis.
- Interventions performed for acute epistaxis should involve packing with material or products that are associated with a low likelihood of rebleeding upon removal, such as lubricated low-pressure pneumatic packing.

**Management of gastrointestinal bleeding:**

- Esophagogastroduodenoscopy should be used as the first-line diagnostic investigation in patients with possible HHT-related bleeding. If colorectal cancer screening criteria are fullfilled and in cases with either genetically proven or suspected SMAD4-HHT syndrome, a colonoscopy should also be performed.
- If the esophagogastroduodenoscopy does not identify significant HHT-related telangiectasia, a videocapsule endoscopy should be taken into consideration.
- HHT-related gastrointestinal bleeding severity should be assessed using the following framework:
  1. Mild: oral iron replacement is sufficient to achieve the target hemoglobin level;
  2. Moderate: hemoglobin goals are met with intravenous iron treatment;
  3. Severe: target hemoglobin levels are not met in spite of adequate iron replacement or blood transfusions are necessary.
- The use of endoscopic argon plasma coagulation during endoscopy should be limited.
- In mild HHT-related gastrointestinal bleeding, the administration of oral antifibrinolytics should be taken into consideration.
- In moderate to severe HHT-related gastrointestinal bleeding, the administration of intravenous systemic antiangiogenic therapies, such as bevacizumab, should be taken into consideration.

**Management of AVMs in children:**

- Diagnostic genetic testing should be performed in asymptomatic children of an affected parent.
- Asymptomatic children with HHT or at risk for HHT at the time of presentation should be screened for brain and pulmonary AVMs.
- Large pulmonary AVMs and those associated with decreased oxygen saturation levels should undergo treatment in an effort to avoid major complications.
- Pulmonary AVM screening in asymptomatic children with definite HHT or at risk for HHT should typically be repeated at 5-year intervals.
- Brain AVMs that harbour high-risk features should be treated.

**Management of anemia and anticoagulation:**

- Iron deficiency and anemia should be investigated in the following HHT patients:
  1. Adults, irrespective of symptoms;
  2. Children presenting with symptoms of anemia and/or recurrent bleeding episodes.
- Iron replacement therapy is recommended as follows:
  1. Oral iron administration constitutes the first therapeutic step;
  2. In cases in which oral administration of iron proves ineffective, cannot be absorbed or tolerated, and in patients presenting with severe anemia, intravenous iron replacement should be performed.
- Red blood cell transfusion is recommended as follows:
  1. Hemodynamic instability or shock;
  2. Comorbidities in which a higher hemoglobin target is needed;
  3. Clinical settings in which an urgent increase in hemoglobin level is necessary (e.g., preoperatively, or during pregnancy);
  4. Inadequate hemoglobin levels in spite of frequent intravenous iron infusions.
- Additional causes of anemia should be investigated in case of an inadequate response to iron replacement.
- Prophylactic or therapeutic anticoagulation or antiplatelets should be administered in the presence of an appropriate indication, taking into account the individualized bleeding risks of HHT patients; HHT-related bleeding does not constitute an absolute contraindication for these medications.
- Dual antiplatelet therapy and/or a combination of antiplatelet medication and anticoagulation should be avoided in HHT when possible.

**Management of AVMs in pregnacy and delivery:**

- Clinicians should address preconception and prenatal diagnostic options in patients with HHT.
- Testing by unenhanced magnetic resonance imaging should be done in pregnant women presenting with symptoms indicative of brain AVMs.
- HHT-affected pregnant women without recent screening and/or treatment for pulmonary AVMs should be managed as follows:
  1. In the absence of symptoms, pulmonary AVMs screening should be performed initially, either by an agitated saline transthoracic contrast echocardiography or by a low-dose chest CT without contrast, according to local availability and expertise. When opting for a chest CT examination, it should ideally be performed early in the second trimester.
  2. In cases presenting with symptoms indicative of pulmonary AVMs, diagnostic testing by a low-dose non-contrast chest CT scan should be performed. This examination can be carried out at any gestational age, as clinically indicated.
  3. The treatment of pulmonary AVMs is recommended starting with the second trimester, in the absence of other clinical contraindications.
- HHT-affected pregnant women with previously untreated pulmonary and/or brain AVMs or those who have not undergone pulmonary AVMs screening recently should be managed by a multidisciplinary team, ideally in a tertiary care center.
- Epidural anesthesia should not be withheld on the basis of a HHT diagnosis and spinal vascular malformations screening is not necessary.
- Labor and vaginal delivery are not contraindicated in pregnant HHT women who harbour objectively defined, non-high-risk brain AVMs. An assisted second stage may be necessary on a case-by-case basis.

Treatment of AVMs (Figure 1) is focused mainly on catheter-guided interventions (embolization or sclerotherapy), but there are also other therapeutic modalities, such as surgery (resection) or stereotactic radiosurgery (using radiation) [21,22].

Embolization therapy is the preferred method as it avoids the risks associated with major surgery, general anesthesia, and loss of pulmonary parenchyma. It has a good success rate, and the most commonly encountered complication is pleuritic chest pain, usually self-limited. Other complications are the migration of the device used in embolization, pulmonary infarction, air embolism in the coronary arteries with consequent angina, and bradycardia [2]. For that reason, a CT scan should be performed 3–6 months after the procedure and then subsequently after 3 years. The contrast echocardiography remains positive in more than 90% of treated patients, in spite of the complete occlusion of visible pulmonary AVMs and is not recommended for follow-up [12].

An association between oral microorganisms and brain abscesses was documented. A very good oral hygiene is indicated along with antibiotic prophylaxis prior to dental procedures associated with risk of bacteremia for patients with pulmonary AVMs. In addition to that, given their high risk of paradoxical embolic stroke and brain abscess, pulmonary AVMs should be treated irrespective of their diameters.

The intravenous administration of bevacizumab should be taken into consideration in cases with symptomatic high-output cardiac failure arrising as a complication of liver AVMs with an insufficient response to first-line treatment [19].

Taking into account the fact that hepatic artery embolization is a temporising intervention with significant morbidity and mortality, it should not be performed in cases with liver AVMs [19]. A referral to evaluation for liver transplantation is recommended in cases of refractory high-output cardiac failure, biliary ischemia, or complicated portal hypertension that arise as complications of liver AVMs [19].

HHT cases that exhibit high-output cardiac failure and pulmonary hypertension should be managed multidisciplinarily by the Center of Excellence for HHT and a HHT cardiologist or a pulmonary hypertension specialty clinic [19].

In the long-term, patients with documented pulmonary AVMs should avoid SCUBA diving in order to avoid their rupture, irrespective of previous treatment. In addition to that, antibiotic prophylaxis should be recommended before procedures associated with the risk of bacteremia and special care should be taken in order to avoid intravenous air when intravenous access is in place [21]. Pulmonary hemorrhage and hemothorax are serious, potentially life-threatening conditions, which can occur as complications of spontaneous pulmonary AVMs rupture. The lifetime prevalence and incidence of these clinical entities in patients with a HHT diagnosis have been found to be 2.7% and 0.16%, respectively [23,24].

Recurrent, spontaneous bleeding is associated with the development of iron deficiency anemia. Epistaxis severity scores correlate with age, hemoglobin, and ferritin levels. HHT patients with genetic mutations (*ACVRL1*, *ENG*, or *SMAD4* mutation) have higher epistaxis severity scores. It seems that anti-fibrinolytics are effective therapies in decreasing epistaxis severity and their long-term use in HHT patients has been proven safe [25]. A recent cross-sectional study performed on HHT patients assessed the effects of on-demand sclerotherapy with polidocanol on epistaxis frequency and severity, with encouraging results. In addition to significantly ameliorating HHT-related epistaxis, this approach also proved highly useful in improving the quality of life [26].

In patients presenting with digestive hemorrhage, the essential role of the gastroenterologist resides in locating the site of the bleeding and, whenever possible, attempting to control it. In this respect, using argon plasma coagulation as an endoscopic therapeutic method could be useful for angiodysplasias located in not only the stomach and colon, but also in the small intestine, which is frequently affected in HHT [27].

A recent international multicentre study evaluating the efficacy and safety of bevacizumab administration in HHT-related bleeding, specifically epistaxis and gastrointestinal hemorrhage, has shown striking improvements irrespective of patient genotype. Treatment with this recombinant humanized monoclonal IgG1 antibody was associated with a significant decrease in bleeding severity and the need for red blood cell/iron transfusion, while at the same time being overall well-tolerated. Therefore, the authors suggest that in cases presenting with severe HHT-related gastrointestinal bleeding or epistaxis, the beneficial effects of this therapy should be taken into consideration [28].

Embolization by interventional radiology remains an essential solution for AVMs [18]. Even in selected cases when surgical procedures are reasonable, establishing the appropriate resection margins usually proves challenging. Moreover, subtotal excisions are typically associated with prompt recurrence and progression: around a 57% recurrence rate within the first year after resection (irrespective of concurrent embolization) and an estimated 86% recurrence rate within one year with embolization alone [18].

Combined therapy (surgical/medical) for complex AVMs is recommended, with recent studies observing a significantly more sustained response in patients who have undergone both medical treatment with sirolimus and embolization [29].

High-dose radiation therapy of an AVM has been shown to induce its obliteration. Radiation beam doses seem to be one of the essential factors that influence the AVMs angiogenesis process following radiosurgery or gamma knife [30].

There is increasing interest in recent literature regarding potential novel therapies for HHT-related bleeding non-responsive to currently recommended treatments. One such example is tamoxifen, a selective estrogen receptor modulator, which has been administered in a transfusion-dependent patient with HHT-related epistaxis and gastrointestinal hemorrhage with favorable outcome and no significant adverse effects [31]. In addition to that, the potential use of the somatostatin analogue octreotide has been investigated in case reports and small series, particularly involving patients suffering from severe gastrointestinal bleeding. The results have been encouraging, as octreotide therapy seems to be effective in these cases, with a good safety profile [32,33]. However, further studies are warranted in order to establish clear clinical indications of these medications.

## 4. Conclusions

Hereditary hemorrhagic telangiectasia is a rare condition, that is usually underdiagnosed. The clinical presentation is typically the result of complications arising from shunting through arterio-venous malformations, such as dyspnea, chronic bleeding, or embolism. It is associated with multiple therapeutic challenges for specialists. Routine screening for AVMs in patients diagnosed with this condition is recommended and can prevent severe complications, such as brain abscesses and strokes. A long-term follow-up should be provided in cases with AVMs, in an effort to diagnose the possible growth of untreated AVMs or the reperfusion of those that have undergone treatment.

In spite of two consensuses, several aspects of AVM screening and management remain controversial even among experts. Multidisciplinary teams are useful in complex cases.

## Data Availability

Not applicable.

## Supplementary Materials

The following supporting information can be downloaded at: https://www.mdpi.com/article/10.3390/jcm11092634/s1. Video S1: Contrast echocardiography with bubbles.
